
JOURNAL INFORMATION
==============================
NLM Title Abbreviation: South Med Rec
NLM Journal ID: 0027317

The Southern Medical Record

Publisher: Biomedical Journal Digitization Project

ARTICLE INFORMATION
==============================
PMCID: PMC9154972
PMID: 36025161
Article version: 1
Subjects: Prescription Department

Prescription Department

Print publication date: 1893 Jul
Volume: 23
Issue: 7
First page: 390
Last page: 391
License: This work is free of known copyright restrictions. For more information, please see PMC Back Issue Digitization.
License URL: https://creativecommons.org/publicdomain/mark/1.0/

==============================

CONSTIPATION.

℞. Mass. hydrarg ......gr. xxiv.
Ex. colocyncth co....gr. xij.
Podophyllin.........gr. iv.
Leptandrin..........gr. vj.
Ex. hyoscyamus......gr. xxiv.
Ex. belladonna.......gr. vj.
Ex. nux vomica......gr. vj.

M. ft. et div. in pil. no. xxiv.
Sig.—Take 1 pill at bedtime.
Dr. Frank Woodbury.

RETENTION OF URINE.

℞. Magnes. sulphat. ....gr. xxx.
Potass. bicarbonat....gr. xx.
Potass. nitratis.......gr. x.
Aquæ...............℥j.

M. Sig.—Take at a draught.—Sir J. Paget, *St. Louis Courier of Medicine*

SOOTHING SYRUP WITHOUT OPIUM.

℞. Oil anise............mxxv.
Alcohol...............℥ij.
Fl. ext. valerian.......℥j.
Oil peppermint.........mxv.
Tinct. camphor........ʒij.
Fl. ext. licorice........℥j.

M. Sig.—Shake the bottle. Dose, ¼ or ½ teaspoonful in water; repeat as needed.—*Med. Age.*

INGROWING TOE NAIL.

℞. Muriatic acid,
Nitric acid.......... aa ʒj.
Chloride zinc............℥j.

M. Sig.—Apply 1 drop to the affected part once a day. This gives instant relief to the pain caused by ingrowing toe-nail.—*The Prescription.*

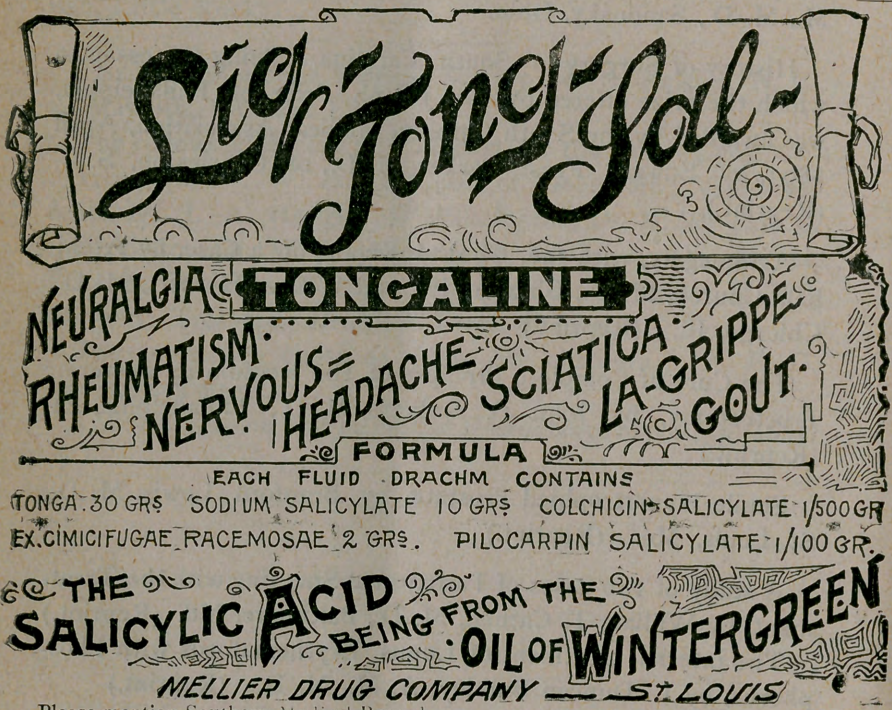

EPIDEMIC CHOLERA.

℞. Opium ..........gr.ij
Gum camphor....gr.ij
Calomel ........gr.iij to vi
Sugar of milk....gr.xv

M. Sig.—Taiturate very thoroughly and administer in a teaspoonful of water.

Dr. A. B. Palmer.

SPRAINS.

℞. Camphoræ..............ʒij
Chloral hydrat ..........ʒiv
Vasalinæ................ʒi

M. Sig.—Rub on to relieve pain in chronic cases.

or :

℞. Ammonii chloridi ........ʒij
Sprts. vini rect ..........℥ij
Aquæ ..................℥ij

M. Sig.—Apply with moist cloth.

TREATMENT OF PAIN IN EYE DISEASE.

When the pain is of corneal origin, Dr. E. Jackson employs one of the two following solutions :

℞. Neutral sulph. atropine.
Muriate of cocaine, a a gr. j.
Distilled water.........ʒij

M. Sig.—Instill one drop of this solution into the eye every four to six hours.

℞. Sulphate of eserine....gr. $\frac{1}{8}$.
Muriate of cocaine ....gr. iss.
Distilled water.........ʒiv.

M. Sig.—Instill a drop into the eye every four to six hours.—*Western Med. Reporter*.

FISSURES OF THE TONGUE.

℞. Acidi carbolici........ʒss.
Tr. iodi.
Glycerini.......a a f ʒiij.

M. Sig.—Use locally.

PURPOSEFUL PRACTICAL.

is ELIXIR THREE CHLORIDES with the small assimilative doses—tonic and alterative—it is designed to meet the very fundamental therapeutic indications, in struma, latent syphilis, malaria, chlorosis, chorea, zymotic, dermatological diseases, etc.—by assailing in a thoroughly scientific manner, the cause.

KINDLY SPECIFY R. & H.'S

ONE BOTTLE ONE MONTH'S REMEDY.

THE MOST WELCOME MEDICAMENT IN GYNAECOLOGY, F. E. POPE, M. D., HONEY GROVE. TEXAS.

FORMULA—Each fluid drachm contains: PROTO-CHLORIDE IRON, 1-8 GRAIN; BICHLORIDE MERCURY, 1-128 GRAIN; CHLORIDE ARSENIC, 1-280 GRAIN; ELIXIR CALISAYA ALKALOIDS.

RENZ & HENRY, CHEMISTS, LOUISVILLE, KY.

INDICATIONS—Struma, Latent Syphilis, General Debility, Habitual Constipation, Loss of Appetite, Tuberculosis, Malaria, Cystitis, Chlorosis, Gleet, Chorea, Zymotic, Catarrhal and Dermatological Diseases, Anemia from any cause, etc.

HENRY'S TRI-IODIDES.

IS A SPECIFIC FOR GOUT AND RHEUMATISM. SEND FOR EVIDENCES OF ITS VALUE.

Please Mention Southern Medical Record.
